# Mildly elevated blood pressure is a marker for better health status in Polish centenarians

**DOI:** 10.1007/s11357-014-9738-9

**Published:** 2014-12-23

**Authors:** Jan Szewieczek, Jan Dulawa, Tomasz Francuz, Katarzyna Legierska, Beata Hornik, Iwona Włodarczyk-Sporek, Magdalena Janusz-Jenczeń, Agnieszka Batko-Szwaczka

**Affiliations:** 1 Department of Geriatrics, School of Health Sciences in Katowice, SUM, Katowice, Poland; 2 Department of Internal Medicine and Metabolic Diseases, School of Health Sciences in Katowice, SUM, Katowice, Poland; 3 Department of Internal Nursing, School of Health Sciences in Katowice, SUM, Katowice, Poland; 4 Department of Biochemistry, School of Medicine in Katowice, SUM, Katowice, Poland

**Keywords:** Centenarians, Blood pressure, Cognitive performance, Physical performance

## Abstract

The number of centenarians is projected to rise rapidly. However, knowledge of evidence-based health care in this group is still poor. Hypertension is the most common condition that leads to multiple organ complications, disability, and premature death. No guidelines for the management of high blood pressure (BP) in centenarians are available. We have performed a cross-sectional study to characterize clinical and functional state of Polish centenarians, with a special focus on BP. The study comprised 86 consecutive 100.9 ± 1.2 years old (mean ± SD) subjects (70 women and 16 men). The assessment included structured interview, physical examination, geriatric functional assessment, resting electrocardiography, and blood and urine sampling. The subjects were followed-up on the phone. Subjects who survived 180 days (83 %) as compared to non-survivors had higher systolic BP (SBP), diastolic BP (DPB), mean arterial pressure (MAP), pulse pressure (PP), higher mini-mental state examination, Barthel Index of Activities of Daily Living and Lawton Instrumental Activities of Daily Living Scale scores, higher serum albumin and calcium levels, and total iron-binding capacity, while lower serum creatinine, cystatin C, folate, and C-reactive protein levels. SBP ≥140 mm Hg, DBP ≥90 mm Hg, MAP ≥100 mm Hg, and PP ≥40 mm Hg were associated with higher 180-day survival probability. Results suggest that mildly elevated blood pressure is a marker for better health status in Polish centenarians.

## Background

The number of oldest-old people, including centenarians, is projected to rise rapidly over the next decades (Kinsella and He 2009). However, knowledge of evidence-based health care in this group is still poor. Hypertension is the most common condition seen in primary care that leads to multiple organ complications, disability, and premature death (James et al. 2014). Guidelines for the management of high blood pressure (BP) in adults define thresholds for pharmacologic treatment with regard to age over 60 and over 80 years (Mancia et al. 2013; James et al. 2014). No guidelines are available for centenarians. Quality of life and a level of independence in activities of daily living rather than life expectancy are matters of the utmost importance at this age. We have performed a cross-sectional study to characterize clinical and functional state of centenarians in Upper Silesia, the most industrialized region of Poland with a special focus on BP. Our preliminary results (Szewieczek et al. 2011) were consistent with those indicating that higher BP was associated with better mental and physical performance in oldest-old people (Nilsson et al. 2007; Euser et al. 2009; Richmond et al. 2011). The paper presents our final results.

## Methods

### Participants

The study was carried out from January 2007 through August 2013. The data regarding hundred-year-old inhabitants of the Voivodeship were obtained from the Silesian Voivodeship Administration Office in Katowice, Poland. According to these data, there were 389 centenarians living in 2006 in the province, accounting for 0.008 % of the total population of the area. Eight percent of addressees responded positively to invitation letters. A visit from the team including a physician and two nurses at the place of residence was scheduled both with the subject and their carer. The study comprised 86 consecutive 100.9 ± 1.2 years old (mean ± SD) subjects (70 women and 16 men).

### Measurements

The assessment included structured interview, physical examination, geriatric functional assessment, resting electrocardiography, and blood and urine sampling. Resting BP was measured at both arms according to the standard protocol. If there was a difference between the arms, the higher value was taken for further analysis. Mean arterial pressure (MAP) was calculated as a sum of diastolic BP (DBP) and 1/3 of the difference between systolic BP (SBP) and DBP. Pulse pressure (PP) was calculated as a difference between SBP and DBP. Ankle-brachial index (ABI) was calculated according to the recommendations of the American Heart Association, with ABI <0.9 indicating lower extremity peripheral arterial disease (Hirsch et al. 2006). Romhilt-Estes electrocardiographic criteria were used to assess left ventricular hypertrophy (Buxton et al. 2006). Mini-mental state examination (MMSE) was used to assess global cognitive performance (Folstein et al. 1975). Katz Index of Independence in Activities of Daily Living (ADL) (Katz et al. 1963), Barthel Index of Activities of Daily Living (Barthel Index) (Mahoney and Barthel 1965) and Lawton Instrumental Activities of Daily Living Scale (IADL) (Lawton and Brody 1969) were used to assess functional status. MMSE was scored from 0 to 30, ADL—from 0 to 6, Barthel Index—from 0 to 100, and IADL—from 9 to 27, with higher scores indicating better functional state.

The following laboratory tests were performed: a complete blood count (erythrocyte count, hemoglobin, leukocytes with differential blood count, platelet count), serum levels of albumin, glucose, insulin, bilirubin, creatinine, folate, vitamin B_12_, cystatin C, total cholesterol, LDL cholesterol, HDL cholesterol, triglyceride, thyrotropin, C-reactive protein, and serum alanine aminotransferase activity. Serum levels of sodium, potassium, calcium, phosphorus (inorganic), iron, and total iron-binding capacity were measured only in 49 subjects. CKD-EPI creatinine-cystatin C equation (Inker et al. 2012) and BIS_creatinine-cystatin C equation (Schaeffner et al. 2012) were used to estimate glomerular filtration rate (eGFR). The methods are recommended in very elderly persons (Lopes et al. 2013). Insulin resistance was calculated according to the Homeostasis Model Assessment (HOMA_IR_) formula (Matthews et al. 1985).

The study subjects were followed up on the phone for at least 180 days after the examination.

Two subjects refused their consent for blood sampling.

### Statistical analysis

The obtained data were analyzed using STATISTICA software version 10 (StatSoft, Inc., USA; StatSoft Poland). Linear and nonlinear regression models were used to assess relationships between selected variables. The Kaplan-Meier method was used to estimate survival probability in subgroups of centenarians distinguished on the basis of different values of the analyzed variables, while differences between these subgroups were assessed with the Wilcoxon-Gehan statistic. The nonparametric Spearman’s rank correlation coefficient was used to assess relationships between functional measures and variables analyzed in the study. Nonparametric Mann-Whitney *U* test was used to compare the data between 180-day survivors and non-survivors. *P* < 0.05 was considered statistically significant.

### Ethics

The study protocol was approved by the Bioethical Committee of the Medical University of Silesia. Written informed consent was obtained from each subject and/or their carer after the aim of the study; protocol and risks were explained.

## Results

**Fig. 1:**
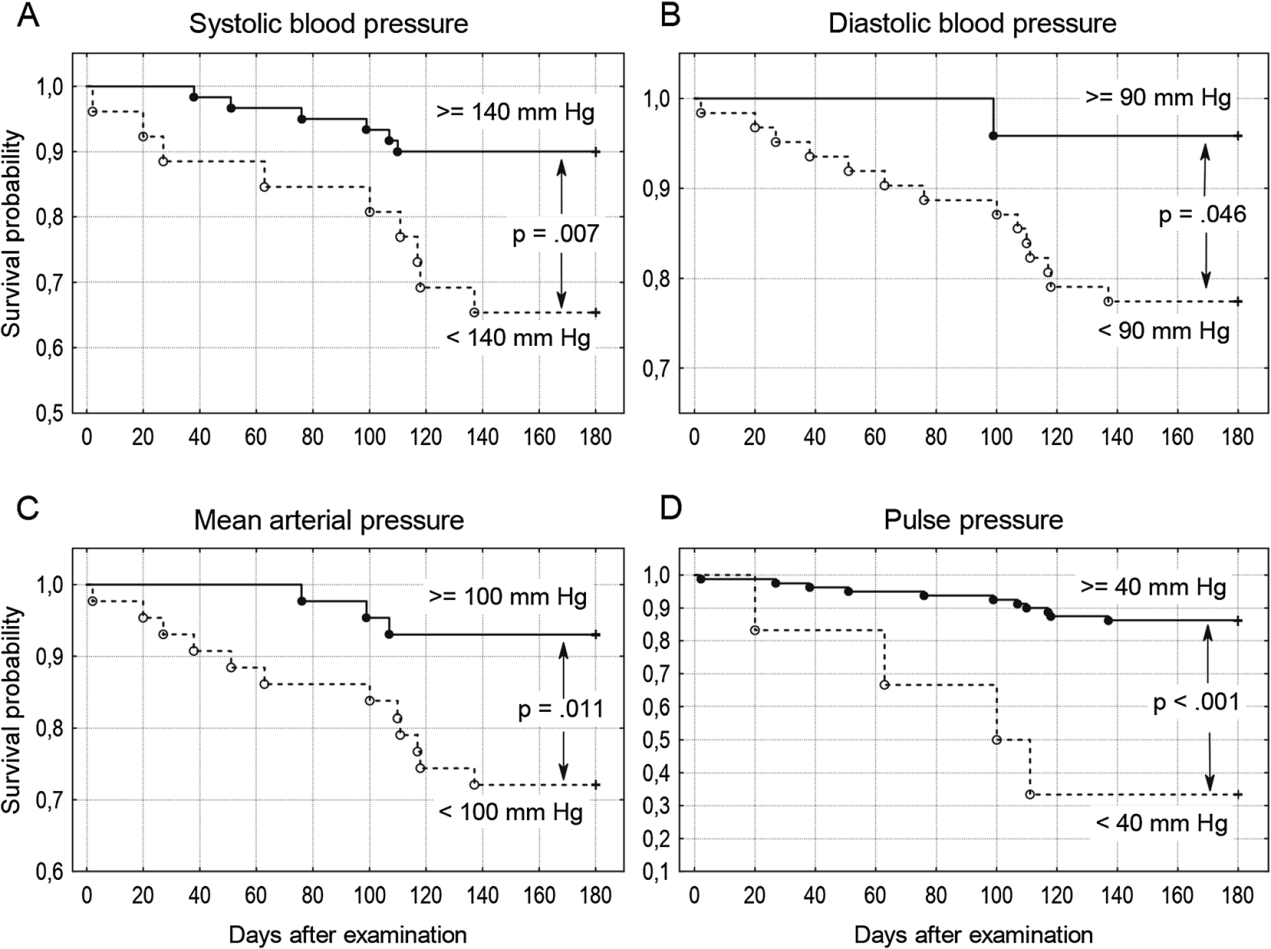
Individuals with SBP ≥140 mm Hg had higher 180-day survival probability (*p* = 0.007), similar to those with DBP ≥90 mm Hg (*p* = 0.046), MAP ≥100 mm Hg (*p* = 0.011), and PP ≥40 mm Hg (*p* < 0.001). **a** Systolic blood pressure. **b** Diastolic blood pressure. **c** Mean arterial pressure. **d** Pulse pressure

**Fig. 2:**
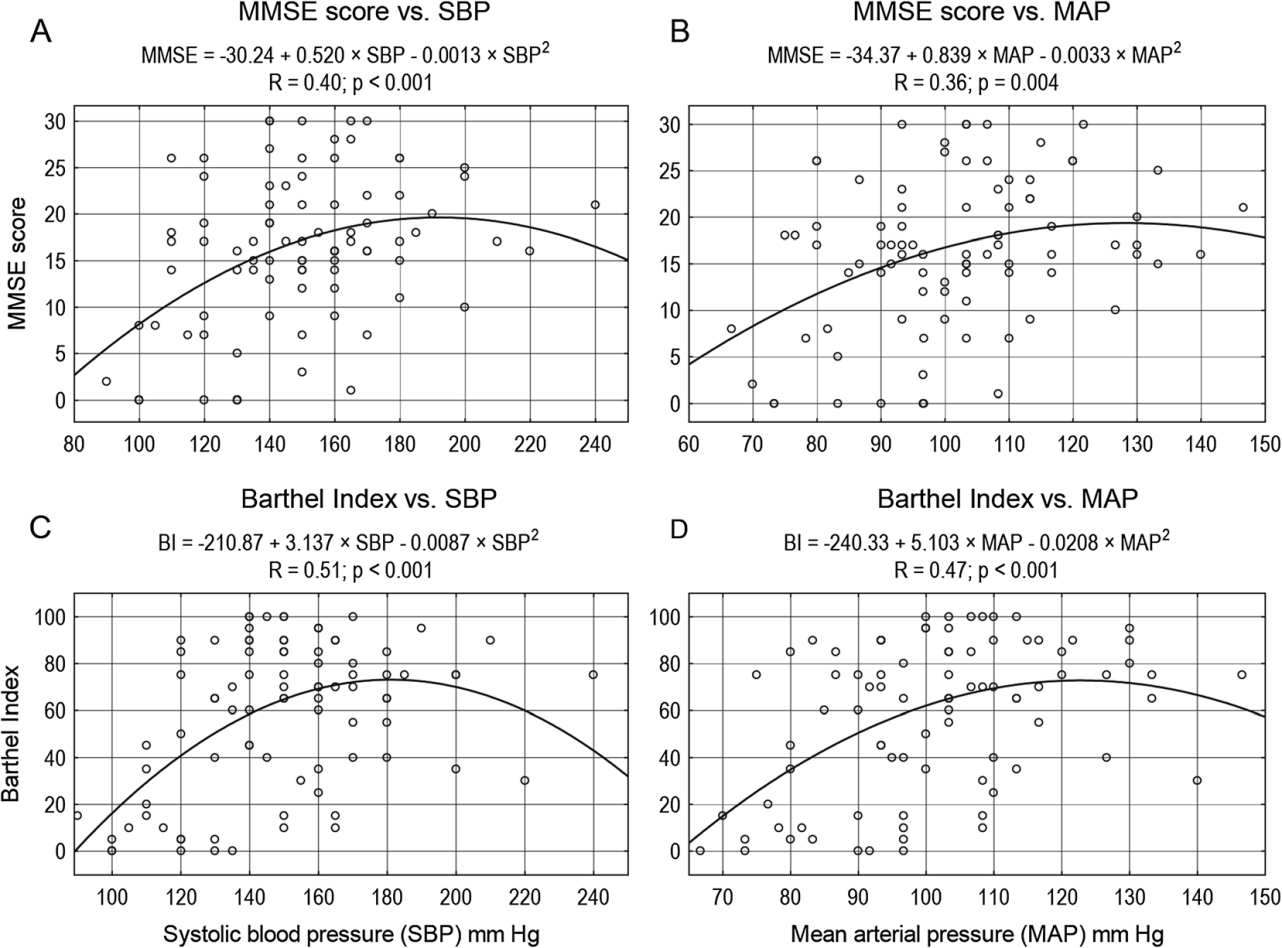
Inverted-U-shaped relationships, according to the second-degree polynomial pattern between MMSE score and SBP, Barthel Index and SBP, MMSE score and MAP, as well as Barthel Index and MAP were revealed. **a** MMSE score vs. SBP. **b** MMSE score vs. MAP. **c** Barthel Index vs. SBP. **d** Barthel Index vs. MAP

**Fig. 3:**
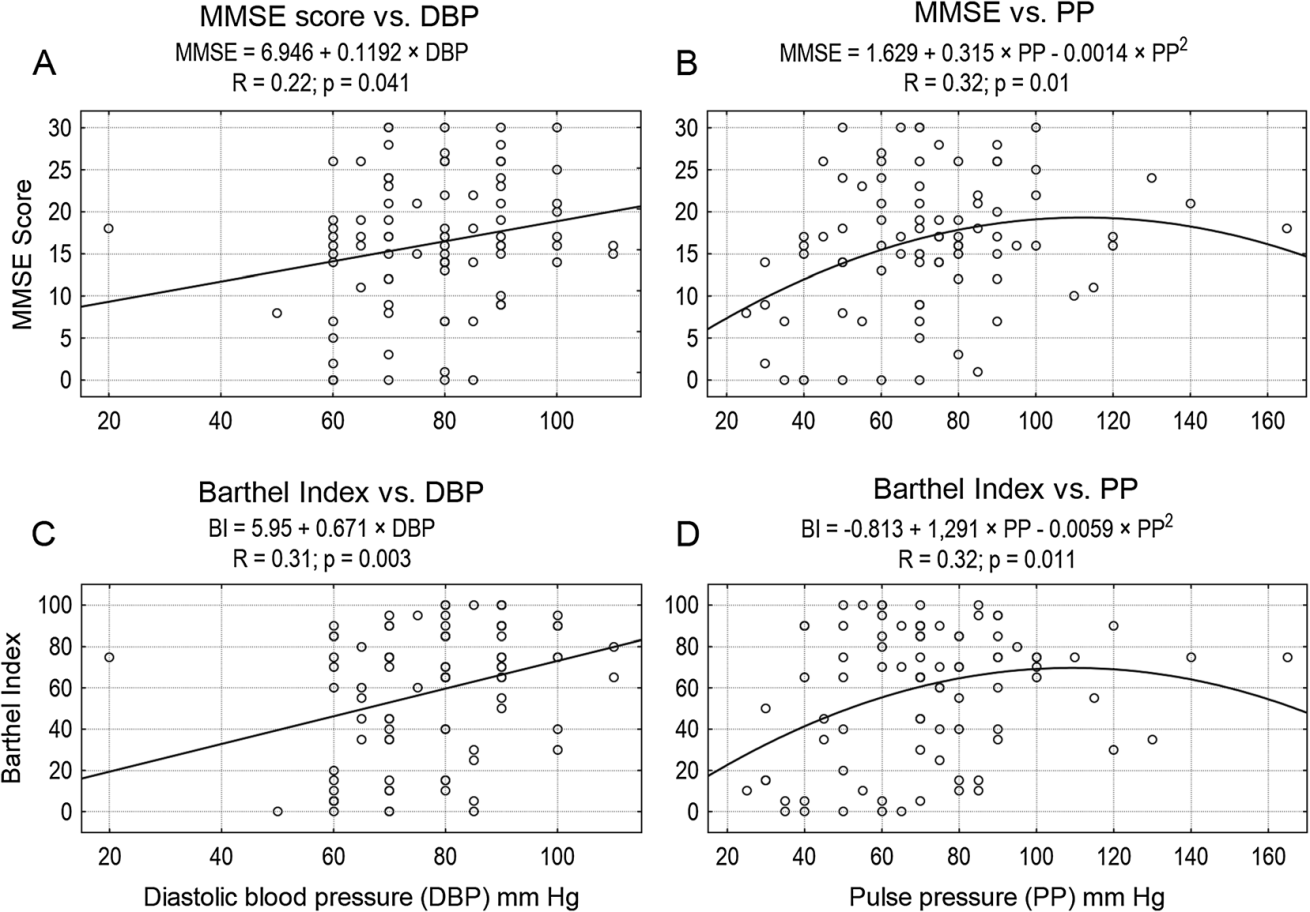
Linear correlation (first-degree polynomial approximation) was the best adjusted model to describe the relationship between MMSE score and DBP, as well as between Barthel Index and DBP, while inverted-U-shaped relationships (second-degree polynomial pattern) between MMSE score and PP as well as Barhel Index and PP were revealed. **a** MMSE score vs. DBP. **b** MMSE score vs. PP. **c** Barthel Index vs. DBP. **d** Barthel Index vs. PP

The most common symptoms reported by the subjects or their carers were as follows: memory disorders (79 %), pain (78 %), and falls (77 %). The most frequent reported diseases were as follows: dementia (73 %), heart failure (65 %), and osteoarthritis (64 %). The majority of subjects (89 %) never smoked. Sixty-three percent of subjects used at least one antihypertensive drug. Characteristics of the group are presented in Table 1. SBP ≥140 mm Hg was found in 69.8 %, and DBP ≥90 mm Hg in 27.9 % of subjects. ABI ≤0.9 was found in 37.5 % of subjects. Seventy-one subjects (83 %) survived 180 days or more. Survivors as compared to non-survivors had higher SBP and MAP values, higher MMSE, ADL, Barthel Index and IADL scores, higher serum level of albumin and calcium, and higher total iron-binding capacity, and lower serum levels of creatinine, cistatin C, folate, and C-reactive protein (Table 1). No significant differences between survivors and non-survivors in blood count, serum levels of glucose, insulin, bilirubin, vitamin B_12_, total cholesterol, LDL cholesterol, HDL cholesterol, triglyceride, thyrotropin, sodium, potassium, phosphorus (inorganic) and iron, as well as in serum alanine aminotransferase activity, eGFR, ABI or Romhilt-Estes ECG scores were found. Individuals with SBP ≥140 mm Hg had higher 180-day survival probability (*p* = 0.007), similar to those with DBP ≥90 mm Hg (*p* = 0.046), MAP ≥100 mm Hg (*p* = 0.011), and PP ≥40 mm Hg (*p* < 0.001) (Fig. 1). SBP ≥145 mm Hg was still associated with higher 180-day survival probability (*p* = 0.042), while SBP ≥150 mm Hg was not associated with significantly higher 180-day survival probability (*p* = 0.068). Inverted-U-shaped relationships, according to the second-degree polynomial pattern between MMSE score and SBP, Barthel Index and SBP, MMSE score and MAP, as well as Barthel Index and MAP were revealed (Fig. 2). Up to the level of around 180 mm Hg, an increase in SBP was associated with an increase in Barthel Index and MMSE score. Similarly, up to the level of 120 mm Hg, an increase in MAP was associated with an increase in Barthel Index and MMSE score. Similarly, second-degree polynomial relationships between MMSE and PP, as well as between Barthel Index and PP were found. Linear correlation (first-degree polynomial approximation) was the best adjusted model to describe the relationship between MMSE score and DBP, as well as between Barthel Index and DBP (Fig. 3). The Spearman’s rank correlation coefficient revealed a relationship between functional measures (Barthel Index, IADL and MMSE), blood pressure, and albumin level in centenarians (Table 2).

**Table 1:** Clinical and functional characteristics of the whole group, 180-day survivors and non-survivors

	All subjects^a^ *n* = 86	Survivors^a^ *n* = 71	Non-survivors^a^ *n* = 15	Survivors vs. non-survivors
Variable	Mean			*p*
Body mass index, kg/m^2^	23.7 ± 4.4	23.8 ± 4.7	23.5 ± 3.2	0.991
Systolic blood pressure, mm Hg	150.0 ± 28.2	153.2 ± 28.4	134.0 ± 22.5	0.015
Diastolic blood pressure, mm Hg	77.3 ± 14.6	78.3 ± 15.3	72.7 ± 10.2	0.123
Mean arterial pressure, mm Hg	101.5 ± 16.5	103.3 ± 17.0	93.1 ± 11.2	0.019
Pulse pressure, mm Hg	72.2 ± 25.2	74.6 ± 25.1	60.7 ± 23.4	0.080
MMSE score	16.2 ± 8.1	17.8 ± 7.2	8.4 ± 7.7	<0.001
Katz ADL	3.21 ± 2.16	3.61 ± 2.02	1.33 ± 1.84	<0.001
Barthel Index	57.8 ± 31.9	63.9 ± 28.4	29.3 ± 33.1	<0.001
Lawton IADL	11.7 ± 4.1	12.2 ± 4.0	9.3 ± 3.5	0.002
Albumin, g/l	37.5 ± 5.5	38.3 ± 5.1	33.5 ± 5.5	0.005
Creatinine, μmol/l	109.1 ± 104.8	100.9 ± 26.2	150.0 ± 253.5	0.044
Cystatin C, ng/ml	1408.5 ± 776.9	1307.6 ± 780.0	1786.7 ± 680.5	0.040
Folate, nmol/l	14.6 ± 9.3	13.5 ± 8.7	19.7 ± 10.8	0.048
C-reactive protein, mg/l	12.1 ± 27.5	8.2 ± 11.4	31.3 ± 60.0	0.017
Total iron binding capacity, μmol/l	51.4 ± 12.8	53.4 ± 10.1	39.8 ± 20.3	0.047
Calcium, mmol/l	2.26 ± 0.14	2.27 ± 0.15	2.20 ± 0.08	0.034
CKD-Epi_creatinine-cystatin C equation, ml/min/1.73 m^2^	42.7 ± 17.9	43.3 ± 18.6	39.8 ± 14.2	0.696
BIS_creatinine-cystatin C equation, ml/min/1.73 m^2^	39.0 ± 17.1	39.7 ± 18.2	35.8 ± 10.2	0.602

^a^Biochemical assays were done in 84 subjects, among them were 70 survivors and 14 non-survivors; serum levels of sodium, potassium, calcium, phosphorus (inorganic), iron, and total iron-binding capacity were measured in 49 subjects, among them were 42 survivors and 7 non-survivors

**Table 2:** Factors correlated with functional capacity measured using the Barthel Index, Lawton Instrumental Activities of Daily Living Scale (IADL), and Mini-Mental State Examination (MMSE) in centenarians

Functional measure	Correlating factors	Spearman’s rank correlation coefficient	*p* value
Barthel Index	IADL	0.696	<0.000
	MMSE score	0.585	0.000
	Systolic blood pressure	0.337	0.002
	Diastolic blood pressure	0.339	0.001
	Mean arterial pressure	0.379	<0.000
IADL	Barthel Index	0.696	<0.000
	MMSE score	0.658	<0.000
	Systolic blood pressure	0.350	0.001
	Diastolic blood pressure	0.308	0.004
	Mean arterial pressure	0.381	<0.000
	Pulse pressure	0.226	0.037
MMSE score	Barthel Index	0.585	<0.000
	IADL	0.658	<0.000
	Systolic blood pressure	0.327	0.002
	Diastolic blood pressure	0.213	0.049
	Mean arterial pressure	0.292	0.006
	Pulse pressure	0.257	0.017
	Albumin level	0.517	<0.000

## Discussion

Hypertension is one of the most common medical conditions leading to multiple cardiovascular complications, disability, and premature death (James et al. 2014). However, substantial differences in the prevalence of hypertension across European countries are observed, from 30 to 45 % of the general population, with a steep increase with aging (Mancia et al. 2013). According to the NATPOL 2011 study, 32 % of adult people in Poland have hypertension (Zdrojewski 2011). PolSenior study revealed that hypertension affects 73.1 % people at the age of 65–69 years, 74.1 % at the age of 85–89 years, and 74.6 % of people at the age of ≥90 years in Poland (Zdrojewski et al. 2012). In our study, the assessment was performed by medical staff at the subject’s place of residence. Some doubts could arise about categorization of such BP measurement. Home BP monitoring refers to BP measurement “away from the medical environment” (Mancia et al. 2013) rather than to BP measurement “at home”. Therefore, we have assumed that BP measurement performed by a doctor at the patient’s place of residence can be considered an equivalent of office BP measurement.

A recent meta-analysis demonstrated that reducing BP to a level of 150/80 mm Hg is associated with large benefit in stroke, cardiovascular and all-cause mortality, as well as heart failure risk in elderly individuals (the mean age of patients on treatment was 71.04 years) (Briasoulis et al. 2014). Many longitudinal and cross-sectional studies demonstrated negative correlation between BP and cognitive performance in middle-aged, young-old, and even old-old individuals (Harrington et al. 2000; Kuo et al. 2005; Knecht et al. 2008; Gamaldo et al. 2008). However, other studies indicated positive correlation between BP and mental or physical performance (reverse epidemiology) in the oldest-old (Nilsson et al. 2007; Molander et al. 2010; Richmond et al. 2011; Sabayan et al. 2012). Waldstein as well as Molander and coworkers found nonlinear relation between SBP and cognitive function with both low and high pressure values associated with poorer results (Waldstein et al. 2005; Molander et al. 2010). Richmond and coworkers found a positive correlation between BP (SBP and DBP) and MMSE scores as well as between BP (SBP and PP) and better functioning on Katz ADL in centenarians (Richmond et al. 2011). Also, our preliminary observations indicated that BP was associated with functional status in a nonlinear fashion with mildly elevated BP (as compared with normal BP defined by threshold values of hypertension (Mancia et al. 2013)) associated with better mental and physical status in Polish centenarians (Szewieczek et al. 2011). Our current analysis confirmed the observations. We have analyzed relationships between functional and clinical measures, as well as between these measures and 180-day survival (Table 1). Measures of mental and physical functional level (MMSE, Katz ADL, Barthel, and Lawton Indexes) appeared to show prognostic factors for survival. On the other hand, they correlated positively to some degree with BP. However, these functional measures were not independent factors associated with BP (Table 2). A recent study on another group of Polish centenarians also revealed that cognitive and functional performances are predictors of survival. In a group of 340 subjects aged 101.4 ± 1.4 years, the mean SBP was 140.7 ± 23.0 and mean DBP 76.4 ± 12.6 mm Hg. PP, SBP, and MAP correlated positively with MMSE, ADL, and IADL score. A higher mean systolic blood pressure and a higher mean pulse pressure were also associated with longer survival, but these associations did not reach the level of significance (Mossakowska et al. 2014). In the Georgia Centenarian Study, the mean SBP and DBP in white centenarians (126.82 ± 14.54 and 72.95 ± 8.99 mm Hg, respectively) were lower than in our group. Hypertension was present in 41 % of those subjects, while mean MMSE (17.4 ± 8.4) was comparable to our group. Data on the association between blood pressure and functional measures are not presented, but facility residents had both lower SBP and lower MMSE (Davey et al. 2010). According to our observations, mildly elevated BP appeared to be a marker of better health (both mental and physical) in the oldest-old, in line with the assumption of Molander and coworkers (Molander et al. 2010). The reason for the association between elevated BP and better functional status in this oldest-old cohort is complex. Firstly, decreasing BP can be a symptom of devastating chronic conditions like Alzheimer’s and vascular dementia (Guo et al. 1998; Qiu et al. 2004) or heart failure (McKee et al. 1971; Poortvliet et al. 2013), especially in individuals with a history of hypertension (Nilsson et al. 2007; Molander et al. 2010: Taylor et al. 2013). Secondly, elevated BP improves organ perfusion (especially in the brain) that worsens with age as a consequence of progressing age- and disease-related vascular changes (Wang et al. 2010). Adequate perfusion is not only critical for concurrent brain function; hypoperfusion can accelerate neural tissue damage (Molander et al. 2010). Thus, mildly elevated BP seems to be beneficial for both mental and physical function in these oldest-old individuals. However, a problem still exists, as hypertension is a risk factor for devastating, potentially fatal cardiovascular events (James et al. 2014). However, our observations indicate that SBP = ≥140 mm Hg, DBP ≥90 mm Hg, MAP ≥100 mm Hg, or PP ≥40 mm Hg are associated with higher probability of 180-day survival in Polish centenarians. The findings are consistent with results of studies which demonstrated that SBP lower than 140 mm Hg in individuals aged 85-plus or SBP lower than 150 mm Hg in nonagenarians is associated with increased mortality (Rastas et al. 2006: Poortvliet et al. 2013). Thus, it may be reasonable to reduce SBP to 150 mm Hg (Mancia et al. 2013; James et al. 2014), but “lower is not always better” in the oldest-old.

Limitations of the presented study encompass low inclusion rate of candidates, causing the observation to be extended in time, a relatively small study group, single BP measurement, and incomplete number of laboratory tests. Telephone-documented 180-day survival defined the life span, whereas the progression of mental and functional disability associated with BP measurements in the surviving centenarians could not be determined due to the lack of the control assessment. Most of the difficulties resulted from the nature of this centenarian cohort. Nevertheless, the observation demonstrated that functional status has higher prognostic value for 180-day survival than any clinical measures, and BP at the level considered as mild hypertension is associated both with better functional status and higher short-term survival probability in Polish centenarians. As it was discussed in our previous paper (Szewieczek et al. 2011), local groups of centenarians can substantially differ. Thus, our conclusion may not necessarily have universal range. BP measurement remains a fundamental method of the medical assessment in centenarians. The question to be solved in further studies is the timepoint when the pharmacological treatment of elevated BP in the oldest-old patients is beneficial.

## Conclusion

Mildly elevated blood pressure is a marker for better health status in Polish centenarians.
